# Exploring Teens as Robot Operators, Users and Witnesses in the Wild

**DOI:** 10.3389/frobt.2020.00005

**Published:** 2020-02-21

**Authors:** Elin A. Björling, Kyle Thomas, Emma J. Rose, Maya Cakmak

**Affiliations:** ^1^Momentary Experience Lab, Human Centered Design and Engineering, University of Washington, Seattle, WA, United States; ^2^School of Interdisciplinary Arts and Sciences, University of Washington Tacoma, Tacoma, WA, United States; ^3^Human-Centered Robotics Lab, Paul G. Allen School of Computer Science & Engineering, University of Washington, Seattle, WA, United States

**Keywords:** social robots, participatory, adolescence, empathy, Wizard of Oz, mental health, human-centered design

## Abstract

As social robots continue to show promise as assistive technologies, the exploration of appropriate and impactful robot behaviors is key to their eventual success. Teens are a unique population given their vulnerability to stress leading to both mental and physical illness. Much of teen stress stems from school, making the school environment an ideal location for a stress reducing technology. The goal of this mixed-methods study was to understand teens' operation of, and responsiveness to, a robot only capable of movement compared to a robot only capable of speech. Stemming from a human-centered approach, we introduce a Participatory Wizard of Oz (PWoz) interaction method that engaged teens as operators, users, and witnesses in a uniquely transparent interaction. In this paper, we illustrate the use of the PWoz interaction method as well as how it helps identify engaging robot interactions. Using this technique, we present results from a study with 62 teens that includes details of the complexity of teen stress and a significant reduction in negative attitudes toward robots after interactions. We analyzed the teens' interactions with both the verbal and non-verbal robots and identified strong themes of (1) authenticity, (2) empathy, (3) emotional engagement, and (4) imperfection creates connection. Finally, we reflect on the benefits and limitations of the PWoz method and our study to identify next steps toward the design and development of our social robot.

## 1. Introduction

Teens are now the most stressed age group, with 27% percent of US teens reporting very high levels of daily stress, and 31% reporting feeling overwhelmed as a result of negative stress (American Psychological Association, 2014). Increased stress has been shown to lead to depression (Maughan et al., 2013) and negatively impacts cognitive function that affecting learning (Vogel and Schwabe, 2016). Many schools lack the resources (time and personnel) to implement and maintain school-based mental health programs (Eiraldi et al., 2015).

Social robots have the potential to improve mental health, especially in teens. Given that teens' lives are mediated through a variety of digital technologies, using a digital device to support them may be contextually appropriate. To address the mental health challenges of teens, Project EMAR (Ecological Momentary Assessment Robot) aims to develop a social robot for teens that will be stationed at schools to gather accurate momentary data about teen stress and provide micro-interventions to reduce stress. We used a participatory design approach, involving teens in all research and design activities and decisions. In the last 3 years, our research team has conducted a number of exploratory high school visits, a social robot design challenge with 7 participating high schools (Rose et al., 2019), and several participatory design and interaction studies that were all in the wild at schools (Rose and Björling, 2017; Björling et al., 2018). Our current investigation involves teens as co-researchers and co-designers to help us explore (1) differences between movement-only and speech-only robot interactions and (2) appropriate robot responsiveness to teen stressors. The goal of conducting this study was to explore both movement and speech behaviors to inform our larger project.

In this paper, we present the results of a study to explore how teens teleoperate and respond to two distinctly different robots. One is a soft-bodied, movement robot with no speech capabilities. The other is an immobile, boxy-robot with speech capability. First, we provide a background on teen stress, social robots, and participatory, human-centered design. Second, we detail the methods of the study and how it was conducted, including the development of a novel method to investigate teen and robot interactions called of participatory Wizard of Oz. Third, we share the findings of the study that explore the complexity of stress, attitudes about robots, comparison of two robot prototypes, and the themes from an analysis of teen engagement with the robot prototypes. Fourth, we discuss the findings and reflect on how the results of the study can inform social robot design for teens. We conclude with a discussion on limitations and next steps.

## 2. Background

### 2.1. Teen Stress and Mental Health

Eighty-three percent of teens report school as a primary negative stressor (Thapar et al., 2012; American Psychological Association, 2014). Recent evidence shows the cumulative impact of everyday sources of negative stress is highly prevalent and impactful on teens (Hamilton et al., 2016). Although positive stress is experienced by adolescents and appears to benefit their well-being (Branson et al., 2019), chronic negative stress is a known risk factor for both physical and mental health problems (Juster et al., 2010) as often stressful life events precede the onset of adolescent depression (Mazurka et al., 2016). The developing adolescent brain makes adolescents especially vulnerable to the cumulative insults of chronic stress (McEwen and Morrison, 2013). A nationwide survey of high school students in the United States found that 16% of students reported that they were seriously considering suicide, 13% reported creating a plan, and 8% reporting trying to take their own life in the 12 months preceding the survey. Adolescents also exhibit the highest rates of self-harm, including attempted suicide (Ting et al., 2012).

Effective, school-based stress reduction interventions for adolescents exist. The Mindfulness-Based Stress Reduction program for teens has been shown to reduce teen stress and decrease the possibility of mental health problems (Biegel et al., 2014; Edwards et al., 2014). In addition, cognitive behavioral and dialectical behavioral school-based therapy programs have both been successful at reducing stress and incidence of depression (Werner-Seidler et al., 2017). However, most school-based interventions are cost-prohibitive and require significant commitment of staff and student time, which is not possible for many schools, especially those that are under-resourced (Eiraldi et al., 2015). Therefore, designing assistive technologies to support teens in reducing stress can result in increased access to necessary mental health tools.

Technologies designed and aimed to reduce stress and improve mental health do exist. Chatbots such as Woebot (Gabriels, 2019) and Vivibot (Greer et al., 2019) have been shown effective in reducing anxiety and depression in adults. And, although a chatbot has been successful in smoking cessation for adolescents (Simon et al., 2019), a large review of chatbots for mental health concluded more reserach is needed to understand the true effect on mental health and none of the chat agents were focussed specifically on stress reduction (Abd-alrazaq et al., 2019).

### 2.2. Assistive Social Robots

Social robots provide a variety of benefits and can assist humans by fulfilling unmet needs (Feil-Seifer and Mataric, 2005). Social robots have been suggested as an appropriate tool for mental health applications, providing therapeutic and assessment capabilities in a variety of populations (Breazeal, 2011) including those that are vulnerable (Kim et al., 2013). Several robots have shown promise in terms of therapeutic interventions. For example, the social robot Therabot (Duckworth et al., 2015) is an animated dog designed to support those who have survived trauma and experience feelings of being overwhelmed. Additionally, Paro (Wada et al., 2005) is a plush seal designed for seniors in assisted living environments to reduce stress and stimulate interaction.

Social robots have also been designed specifically for their therapeutic effect for children. Researchers have identified the importance of empathy in social robot interactions with children (Leite et al., 2012; Giannopulu et al., 2018). Social robots have been highly effective for increasing social interactions and communication for children with autism (Fernaeus et al., 2010; Scassellati et al., 2012; Kim et al., 2013). In addition, social robots have been shown to reduce anxiety in children who are hospitalized (Jeong, 2017; Logan et al., 2019). Little work has specifically explored the relationship between teens and social robots.

The design of a social robot to specifically help measure and address teen stress, is a timely expansion of the application of social robots with potential for significant benefits. Project EMAR is an interdisciplinary project using human-centered design to develop a social robot to capture stress and mood data from teens while providing a micro-intervention to relieve stress. EMAR is designed to live in a school setting and collect aggregate, anonymous data and be a tool for teens to better understand and manage their stress, see Björling et al. (2018) for more detail.

### 2.3. Participatory, Human-Centered Design With Teens

In designing and developing social robots, our project uses human-centered design (HCD), an approach to developing technology that focuses on people and their needs throughout the design process, defined by ISO 9241-210:2010(E). It is a process with a philosophical commitment to upholding human dignity and human rights (Buchanan, 2001; Walton, 2016). Within HCD, this project employs participatory design methods to engage participants (Schuler and Namioka, 1993) which is an appropriate way to engage vulnerable populations such as teens.

While the methods for this study are detailed further in the methods section below, we differentiate our approach from other common approaches to designing social robots. First, the research in this study is conducted in the wild, rather than in a lab. Lab studies can not adequately account for the open-ended encounters that happen between people and robots that are context-dependent (Šabanović et al., 2006). Studies in HRI often privilege the technological capabilities of robots over important factors such as social context and needs of a diverse group of users or stakeholders (Šabanović et al., 2014).

A variety of methods are appropriate for engaging people in design in the wild. Each of these methods have strengths and weaknesses. Contextual inquiry (Beyer and Holtzblatt, 1998) is a method where the researcher engages in detailed observation and interviewing in the context where a product or design will be used, in the wild. This method places the researchers in an apprentice relationship and privileges the expertise and perspective of the target user. However, while this method is well suited to gaining an understanding of how existing processes and procedures, specifically in work settings are completed, it is less appropriate for groups of people, engaging in loosely structured interactions with novel technologies in a social setting. Further, ethnography is a helpful method for understanding a culture or group of people as a way to inform design (Millen, 2000; Olson and Kellogg, 2014). Ethnography is helpful to understand the use and adoption of novel technologies, including social robots, over time (Forlizzi, 2007; Sabelli et al., 2011). However, ethnography is not always an appropriate method choice when designing new or novel technologies that are not fully functional and still require formative feedback and iteration.

Given our participatory approach, we were primarily interested in choosing methods that specifically engaged teens as collaborators in both the research and design process. Other methods that recognize the expertise of users, include the approach of Ladner (2015) to design for user empowerment which calls on the HCI community to build infrastructure and design opportunities to promote the ability for more people to be engaged during the end to end process of design. As he states, “In design for user empowerment, users develop the project, design the requirements and features, develop the prototypes, test the prototypes, and analyze the results of testing to refine the design” (p. 27). Engaging people throughout design in a meaningful and fully engaged way can create more appropriate design solutions.

Other approaches that engage users in the design of social robots that comes closer to Ladner's vision, includes the technique of body storming, where one person role plays being a robot in order to explore design ideas, interactions, and scripts (Oulasvirta et al., 2003). An additional method that includes even more interactivity from this type of role-play is the Wizard of Oz technique, a common approach for simulating the functionality of design (Kelley, 1984) where an operator simulates key features of a technology. The challenge of the Wizard of Oz approach is that it often includes deception and is not transparent about what aspects of the technology are simulated or functional. The Wizard of Oz technique has not been explored extensively in participatory design, with the exception of one study asking participants to create their own gestural interfaces using the Wizard of Oz technique (Akers, 2006).

Participatory Design (PD) has been used in other contexts to develop social robots. Many robotics projects start with a technological stance or intervention, whereas PD starts with an aggregation and analysis of the concerns of a community or group. Members of that group become active in the design process throughout the project. Designs are synthesized from that stand point rather than exclusively gathering feedback on existing designs (Rodil et al., 2018). PD invites participants to use their experiences and bring “their lifeworlds through their design” (DiSalvo et al., 2008; Rodil et al., 2018) and engage in “critical engagements” that can reveal and question existing beliefs about technology (DiSalvo et al., 2008).

## 3. Study Design: Exploring Different Types of Robots During Teen-Robot Interaction

The long term goal of our research is to design and develop a social robot that can capture and aggregate data about perceived adolescent stress in schools and offer interventions to help to reduce teen stress. In the past 3 years, we have gathered input from teens to inform the design using a number of different methods. In this paper, we focus on the context of teenagers talking to the robot (in free form) to share details of what stresses them (e.g., an upsetting interaction or an upcoming exam). Our goal was to better understand how the robot should behave such that users feel heard. In particular, we sought to gather input about different embodiments (section 3.2) and understand parameters of robot behaviors (how it should move, what it should say) to give the sense of being heard. In the following section, we describe our method for gathering data from teens in the wild (i.e., at schools), describe the procedure we followed, and enumerate the types of data we gathered.

### 3.1. Participatory Wizard of Oz Method

While the advantages of observing human-robot interactions in the wild are clear, the researchers' ability to do so is often limited by the availability of social robot platforms that allow rapid prototyping of robust interactions. To address this challenge and take full advantage of conducting studies in the wild with teen participation, we developed a new method that extends the Wizard of Oz technique. Our method, which we call *Participatory Wizard of Oz*, involved removing the typical deception of the WoZ method where a researcher operated the robot without the participant's awareness. Similar to the suggested framework of Druin (2002) where child participants were placed in multiple roles, e.g., user, tester, informant, or design partner, our Participatory WoZ (PWoZ) method was fully transparent with the following characteristics:

- Research was conducted in the wild (*in situ*).
- Participants were the creators of interaction content.
- Participants were wizards of the robot.
- Participants were users of the robot.
- Participants were witnesses to the robot interaction.

**Figure 1:**
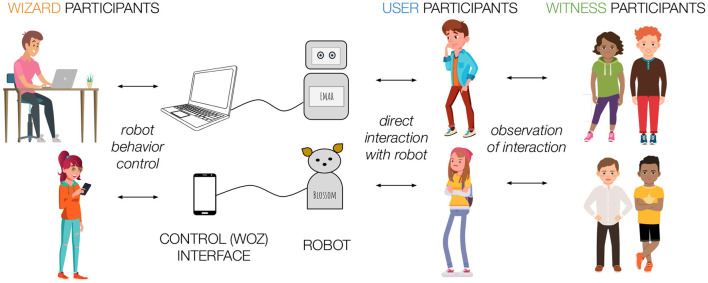
Illustration of the Participatory Wizard of Oz in the Wild Method used in our research. Wizards control robot behavior, users directly interact with the robot, and witnesses observe user-robot interaction.

The characteristics are illustrated in Figure 1 and described in more detail below.

#### 3.1.1. In the Wild

Conducting research “in the wild” is critical to the goal of developing a social robot that will ultimately be implemented in schools and will require continued engagement from teens in order to have an impact on helping them cope with stress. Having teens interact with our robot prototype in the same context in which the robot will be implemented, enabled them to consider environmental contexts that may not be evident in a lab setting. Further, maintaining ecological validity (Oulasvirta et al., 2003; Carter et al., 2008), greatly strengthened our data stemming from this PWoZ method. Unlike laboratory studies, our study embraces the numerous, uncontrollable variables that exist in the wild. We allow for freedom of choice, the influence of social factors and interactions, and real world distractions. We utilized real-world spaces to conduct our studies in the wild (e.g., flexible spaces and classrooms). In addition, researchers stepped out of the way during interactions and were often not visible, making the interactions even more contextually valid. Finally, studying the interaction in context gave us the opportunity to study how the interaction might be perceived by observers.

#### 3.1.2. Four Participant Roles

In this method, participants played all the key roles in the social interaction, and provided data about their experience of each role including (1) content creators, (2) robot operators (Wizards), (3) robot users, and (4) interaction witnesses. By asking participants to fulfill each of these roles, researchers primarily became facilitators of the method, rather than, wizards or witnesses, therefore allowing teens to be more naturalistic in their group interactions. Researchers set up the study and provided an overview, but faded into the background of the research context as teens design and drove the interactions.

In this method, teens participated in the research in multiple capacities providing design input and feedback from different perspectives. Wizard participants provided input about how they thought the robot should behave by controlling the robot's actions, such as what the robot said, what facial expression it displayed, or how it moved in reaction to the user's story. User participants directly interacted with the robot prototype demonstrating how the interaction unfolded and gaining first hand experience about how the interaction felt. Witness participants observed the interaction from a third person perspective. After experiencing the interaction from three different perspectives, participants in all three roles provided feedback about the robot's behavior in the interaction (chosen by the wizard participant) as well as attributes (e.g., size, material, look) and capabilities (e.g., voice, range of motion) of the robot.

This method combined advantages of many alternative methods discussed in section 2.3: (1) in the wild, (2) low fidelity prototype (like WoZ), (3) significant involvement of teens in multiple roles. One clear distinction of this method from the traditional Wizard of Oz method was that the user participants who interacted with the robot were not under the impression that the robot is operating autonomously. Teens were fully aware that the robot was controlled by their peer, making the experience more teen-centric.

### 3.2. Robot Platforms

**Figure 2:**
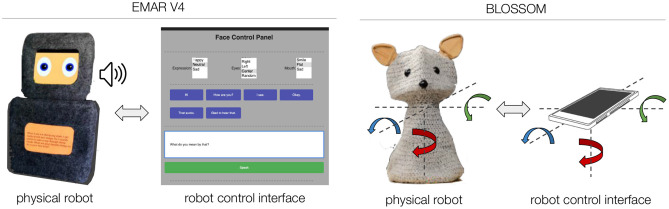
The two robot platforms used in our studies. **(Left)** EMAR V4 and the browser-based control interface for making the robot speak and changing its facial expression. **(Right)** Blossom and the mobile phone interface for controlling the robot's pose and movement.

Our prior research with teens resulted in a number of design requirements, but due to the variability we observed in teens' preferences we had not yet fully committed to a particular robot platform. In this work, we explored the use of two different robot platforms, both with high degrees of customizability. Given we wanted to explore the functions of speech and movement, the robots were chosen given their specific functionalities (speech or movement) and limitations (lack of speech or movement). In addition, both of these platforms had simple and intuitive control interfaces, which was key to enabling our research method in which teens take the role of wizard to control the robot. The two platforms and their control interfaces are shown in Figure 2.

**EMAR V4** is a social robot designed for facial expression and speech communication. It has a box-like structure based upon previous design requirements from teens (Björling et al., 2018). It has two Nexus 7 tablets encased in a soft felt body. One tablet is used as the robot's face, which is a web application running on a browser on the tablet. The face has two eyes that blink and its facial expression can be changed. This tablet is also used to make the robot speak using the browser's text-to-speech capability. The other tablet is located at the robot's belly and is intended as an input/output touchscreen for communication with the user.

The robot's actions are controlled through another browser-based “Wizard of Oz” interface. In this study, the primary way in which the robot responded to the user was through speech. The control interface has a small number of buttons corresponding to simple pre-specified utterances (e.g., “I see,” “That sucks”) that the wizard can trigger in response to the user's utterances. The interface also includes a free form text box that the wizard can type in what they want the robot to say. In addition to making the robot speak, the wizard had control over what facial expression the robot displayed (neutral, happy, sad) and where the robot looked (center, up, down, left, right, randomized). Components of the robot (two tablets) and the control interface communicated through a real-time database. The robot can be customized in different ways such as changing features of the face, using shells of different size, color, or material, and dressing the robot with additional accessories.

**Blossom** is a soft-bodied, flexible robot with a crocheted outer shell and a 3 degrees of freedom inner mechanism (Suguitan and Hoffman, 2018). The robot has no facial expression or speech capabilities and therefore represents movement as its only response. The mechanism allows the robot to rotate around the vertical axis (pan) and bend its neck down in any direction (tilt).

Blossom is teleoperated in real time using a smartphone with a gyroscope and magnetoscope. The pan/tilt angles of the smartphone determined by these sensors are directly mapped to the robot's neck pan-tilt angles. This is done in a tight loop that enables continuous motions of the robot to be transformed into continuous robot motions such as nodding or shaking the robot's head.

## 4. Methods

### 4.1. Sample

**Figure 3:**
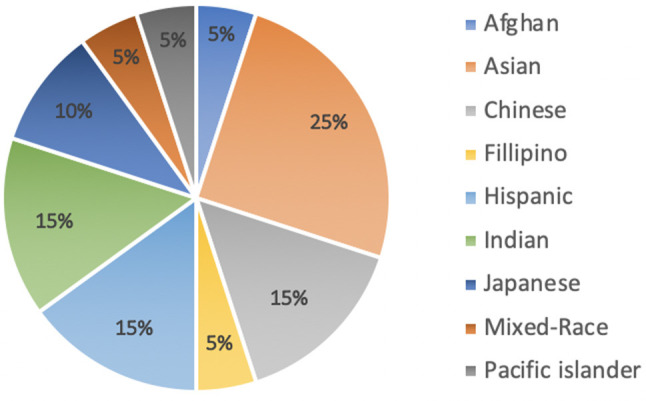
Self-reported ethnicities of our non-white participants (*n* = 22).

The study was conducted in four Pacific Northwest urban, public high schools. Teens were recruited from a physics class, a computer science class, an after school STEM club, and a Girls Who Code club. Participants were asked demographic questions including age, grade, and self-reported gender and ethnicity. No identifying information (names or contact information) was gathered. We captured data from 62 teens between the ages of 14 and 18 (*M* = 16.77) and in grades 9–12 (*M* = 11.13), see Table 1. Twenty-four females, 32 males, and 5 teens who identified as non-binary, participated in our interaction study. Teens were invited to self-identify their ethnicity in an open question. See Figure 3 for a summary of reported ethnicities.

**Table 1:** Participant ages and grade levels.

**School**	***n***	**Age (m)**	**Grade (m)**
1	23	17.61	11.96
2	20	16.07	11.96
3	14	16.25	10.58
4	5	17.00	11.00

### 4.2. Instruments

#### 4.2.1. Negative Stress

It was important to understand teens' stress levels. Therefore, self-reported stress reflecting on the past month was captured using the Perceived Stress Scale (Cohen and Williamson, 1988) as part of our intake questionnaire. The PSS is a 10-item questionnaire that measures the degree to which situations in one's life are appraised as stressful.

#### 4.2.2. Robot Attitudes

In order to capture teens' beliefs about robots, participants completed a slightly modified, 10-item version of the Negative Attitudes Toward Robots Scale (NARS) (Nomura et al., 2006). NARS has been used in many experiments to evaluate participant attitudes toward many kind of robots. It consists of three subscales:

**S1:** Negative Attitude toward Situations and Interaction with Robots (6 items).
**S2:** Negative Attitude toward Social Influence of Robots (5 items).
**S3:** Negative Attitude toward Emotions in Interaction with Robots (3 items).

To make NARS appropriate for teenagers we removed questions that were written from an adult's perspective, such as “I am afraid that robots may negatively influence children's mind” (S2) and “I feel anxiety when I imagine that I may be employed and assigned to a workplace where robots should be used” (S1). We retained all of S3 as we were most interested in teen's attitudes related to emotions in robot interactions. We also added three new items related sharing data with robots and the general role of robots (Q8–Q10 in Table 2). We used the standard NARS 5-point Likert scale from Strongly Disagree (1) to Strongly Agree (5) for all items.

**Table 2:** Baseline measurement of teen attitudes toward robots.

		**Question**	**Mean**	***SD***
Q1	NARS	I would feel uneasy if robots really had emotions.	2.69	1.16
Q2^*^	NARS	I would feel relaxed talking with robots.	3.26	1.08
Q3^*^	NARS	If robots had emotions, I would be able to make friends with them.	3.23	1.19
Q4^*^	NARS	I feel comforted being with robots that have emotions.	2.74	0.981
Q5	NARS	**I would feel very nervous just standing in front of a robot**.	**4.02**	1.09
Q6	NARS	I would feel nervous talking with a robot in front of other people.	3.11	1.24
**Q7**	NARS	**I would feel paranoid talking with a robot**.	**3.54**	1.12
Q8^*^		I would trust a robot with my data.	2.57	1.10
Q9^*^		I would feel comfortable sharing my emotional data with a robot.	2.98	1.18
Q10^*^		**I think robots can help people**.	**4.46**	0.91

* *Indicates reverse coded. There were n = 61 teen participants who completed the attitude survey before participating in robot design activities. Items where most participants selected “Strongly Disagree” or “Strongly Agree” (reverse coded) are in bold*.

#### 4.2.3. Interaction Survey

We created a brief survey to capture data from the PWoz interactions. We asked wizards, users and witnesses to respond to the survey after each teen-robot interaction activity. The PWoz survey consisted of a brief 7-point Likert scale in response ranging from 1 = not at all to 7 = very much to two items about the interaction for the users and the witnesses. For the operator, we asked an open-ended question shown below which led to descriptions regarding how they tried to operate the robot to show it was listening.

- Operator: How did you try to communicate that the robot was listening? (open-ended).
- Users: How much do you think the robot was listening to your stress story? (7-point Likert).
- Witnesses: How much do you think the robot was listening to the speaker? (7-point Likert).

#### 4.2.4. Exit Interview

The exit interview was a customized, single question prompt with probes targeted toward concerns teens may have about the robot. “If a robot were in your school to help with stress, what concerns might you have?”

### 4.3. Ethics

The research was reviewed and approved by university Internal Review Board and school district research review. Students who were under 18 also obtained parental permission for their participation in our research study. No personal identifiers were captured during the study, only study ID numbers were assigned to identify participants. Photos and videos were taken for research purposes and parents and teens had the option to also opt in to give permission for the photos to be used for social media and research publications. Given the importance of maintaining trusting relationships with teens (Björling and Rose, 2019) and their school communities, no deception was used in our study. In fact, teens were told up front about our research and our project, our intention for this particular study, as well as our process of using a participatory, human-centered design approach. Teens were made aware of how the robots were programmed and operated. All teens had the option to opt out of any activity at any time. A few teens refused or forgot to complete a survey, but all teens engaged in the interactions and often seemed disappointed when the activity was over. No personal data (names or contact information) were captured at any time. Teens were assured their video and interaction data would be used for research purposes only. Data were stored in password protected and university approved online database and were accessible only to the research team.

### 4.4. Study Procedure

The following section describes the study procedures and data collected as part of the study.

#### 4.4.1. Introduction to the Study

All studies were conducted in high school classrooms. We arrived and set up multiple stations including multiple versions of V4 and Blossom with one researcher facilitating the interactions for each station. Before beginning, we presented an overview of the project to the whole group as well as provided some background on the process of human-centered design. Teens were reminded of the consent process and their option to disengage at any time during any of the activities.

#### 4.4.2. Questionnaires, Scripts, and Storyboards

Teens completed intake questionnaires (Demographics, NARS, PSS) and were then divided into pairs to create a stress story (either a script or a storyboard scenario). Teens collaborated together to create these materials for use in the study. We observed that this collaboration was engaging for teens and elicited a great deal of data as teens worked together to illustrate their experiences of stress.

#### 4.4.3. Teen-Robot Interaction Activity

After completing stress stories, teens were assigned to groups of 3-4 and were directed to interaction activity stations. The number of groups was dependent upon the sample at the site which can be referenced in Table 1. At larger sites (schools 1 and 2), teen groups were randomly assigned to an interaction station with either Blossom or V4. At smaller sites (schools 3 and 4), teens had time to interact with each of the robots.

At their interaction station, teens received a brief overview of the platform (V4 or Blossom) and how it is controlled. They chose a role (user, wizard, witness) and were assured they could alternate roles if desired. The wizard was shown how to control the platform and decided upon a comfortable position for operation. The wizard was often visible to the user during interaction. The user then chose to share their own stress story, or a script of other stories written by teens. Witnesses were given seats where they could witness the entire interaction. Once the teens were ready for their roles, the researcher started video recording and moved away and observed from a distance to help the teens to feel comfortable. At the end of the interaction, the researcher returned and handed each participant the brief PWoZ questionnaire. Then teens then had the option to change roles to experience another side of the interaction. All groups were able to rotate roles at least once, offering each teen at least two different roles.

#### 4.4.4. Wrap Up and Group Interviews

After the teen-robot interaction activities, the group came back together to complete a final NARS questionnaire. All data were collected and then teens were broken into groups of 4–7 for a group interview. During the group interview, researchers asked questions about their experience and opinions of the robots. Finally, the teens were offered a chance to ask any questions about the study or the robots and given robot stickers as a thank you for their time.

### 4.5. Analysis

Both the NARS and the PSS data were reverse coded appropriately and then scored. Descriptive analysis was used to explore total scores and individual items. Statistical normality tests were performed. In addition, a repeated measures *t*-test in SPSS, version 24 was used to detect any differences in the pre and post NARS total or individual items. A one-way ANOVA was used to explore differences in both the NARS and PSS in relation to grade, age, or gender. Likert scale responses from interaction witnesses and users were analyzed using independent samples *t*-tests to determine any differences between the two robots, EMAR V4 and Blossom.

Exit interviews, storyboards, video interaction data, and open ended responses from wizards were explored qualitatively using a collaborative, applied thematic analysis (Guest et al., 2011). The team of four authors divided data sources by study site and began with review and immersion into the raw data. Using the method of open coding and extraction of salient excerpts, the team made an effort to maintain the context of extracted quotations. The team used the collaborative, online Miro.com (Miro, 2019) site to capture data excerpts including video clips, text and images. The team then met to explore all of the extracted data, the associated context, and preliminary, emergent codes. Through this discussion, substantive themes were collaboratively identified and documented along with their associated evidence. Each author then used a priori coding method on the data again to explore further confirmation of existing themes.

## 5. Findings

### 5.1. Complexity of Stress

**Figure 4:**
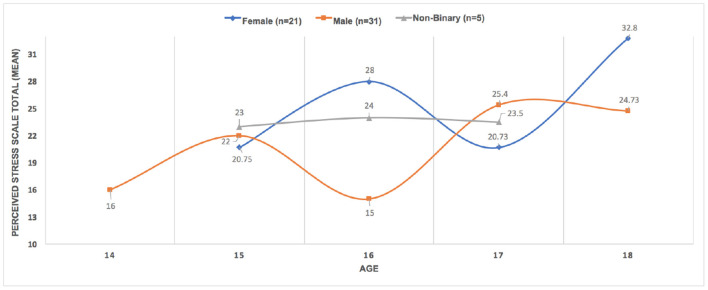
Gender differences in participant mean stress scores.

Given our larger project's aim to design a robot appropriate to both gather stress data, while providing a micro intervention, it is important to capture the general stress level of our teen co-designers. As has been found previously (Björling and Singh, 2017; Björling et al., 2019), the teens in this particular study had high to very high stress levels as indicated by the Perceived Stress Scale (PSS) instrument. As mentioned in the instrumentation section, the PSS is a retrospective self-report referencing the individual's past month. These data allow us to understand the current context in which our participants are interacting with the robots. The participants' mean stress score of 23.16 (SD 6.67) was much higher than the PSS published norm (*m* = 14.2). Nineteen percent of teens (*n* = 12) scored at the low stress level, 67.7% (*n* = 42) scored at the moderate level, and 9.7% (*n* = 6) scored at the high level. Stress scores did not significantly differ by age or grade, although they were higher for teens in 11th grade. See Figure 4 for more detail. However, PSS scores were higher for females (*m* = 21.09), compared to males (*m* = 17.76), and significantly higher for participants who identified as non-binary/fluid (*m* = 23.25) [*F* = 3.321 (*df* = 2) *p* = 0.043]. However, it is important to note that that the non-binary/fluid group was very small consisting of only five participants.

Stress was a ubiquitous experience among all the teens in the study. They had no trouble illustrating stress stories or storyboards depicting their recent or common experiences of stress. Teen stories of stress typically illustrated academic stress, commonly related to grades, test scores, or college. Their stress stories illustrated the breadth of their stressors including, relationships, financial worries, and feeling alone. As one teen stress story illustrated, “I just feel like nobody cares about my problems” [Group activity, School 1]. Stress stories included experiences of feeling pressure from teachers, coaches and parents, e.g., “The pressure Ted had been receiving from his father figure was dampening his whole life.” [Group storyboard, School 1] Teen stress stories also illustrated the experience of competing priorities, typically described as academic and extracurricular (sports) or paid work. For example, “Sam feels super stressed knowing he has to study for all of his tests while juggling the rest of his life. He has sports practice each day and doesn't know how he'll be able to make everything work” [Group storyboard, School 1].

Finally, some outliers were illustrated in the teen stress stories including the articulation of being stressed about a “sexist teacher” and not knowing how to handle the situation. “There is a very sexist teacher named X at school, but when Hank confronts him, he implies that Hank is stupid…Hank doesn't know what to do” [Group storyboard, School 1].

Teens also shared reasoning for why they are not sharing their stressors with friends and family. One female teen stated, “I know I should be able to talk to people, but I don't want to disappoint anyone” [Group storyboard, School 1]. Another male teen verbally expressed that it is often difficult to talk about stressors with friends and family as often they are part of what is creating stress [Group interview, School 1].

### 5.2. Attitudes Toward Robots

Total NARS scores (*m* = 32.61) were not significantly different by age. However, similar to the stress scores, the NARS scores did differ significantly by gender with the highest mean score reported by participants who identified as non-binary/fluid (*m* = 38.5) followed by males (*m* = 33.3), and then females (*m* = 30.70) [*F* = 3.35 (*df* = 2), *p* = 0.042]. As far as items on the NARS, most teens strongly disagreed with feeling nervous about standing in front of a robot or talking to a robot. They also felt strongly that robots could help people. See Table 2 for more detail.

After the teens interacted with the robots in our study as wizards, users, and witnesses, their overall negative attitude scores decreased significantly. Forty-seven participants fully completed all 10 items of the NARS before and after interacting with or operating robots. Significant differences were found including: (1) decreased uneasiness in talking to robots, (2) increased comfort with robots who have emotions, (3) and increased belief that robots can help people. Strongly scored items at intake such as disagreement with the statement about feeling paranoid talking with a robot, and nervousness standing in front of a robot remained stable. See Table 3 for more detail.

**Table 3:** Participant change in NARS item.

**Paired differences**	***n***	**m (diff)**	***SD***	***SE***	***t***	***df***	**Sig. (2-tailed)**
**Uneasy**	**49**	**−0.469**	**0.915**	**0.131**	**−3.59**	**48**	**0.001**
Relaxed	49	−0.18367	1.01393	0.14485	−1.268	48	0.211
Friends	49	−0.16327	0.74574	0.10653	1.533	48	0.132
**Comforted**	**49**	**−0.26531**	**0.83605**	**0.11944**	**−2.221**	**48**	**0.031**
Nervous Standing	49	−0.224	0.985	0.141	−1.596	48	0.117
Nervous Talking	49	0.102	1.388	0.198	0.515	48	0.609
Paranoid	48	−0.083	0.986	0.142	−0.586	47	0.561
Trust	49	0.12245	0.9494	0.13563	0.903	48	0.371
Sharing Emotion	48	−0.16667	1.01758	0.14687	−1.135	47	0.262
**Robots Help**	**49**	**−0.32653**	**0.94401**	**0.13486**	**−2.421**	**48**	**0.019**
**NARS Total**	**47**	**−1.382979**	**3.892876**	**0.567834**	**−2.436**	**48**	**0.019**

*Statistically significant items are in bold*.

### 5.3. Comparing EMAR V4 Blossom

**Figure 5:**
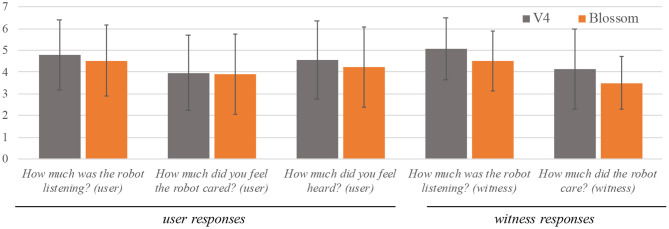
Post-interaction survey responses comparing EMAR V4 and blossom.

A key intention of our study was to explore differences between teen responsiveness to a non-moving, verbal robot (EMAR V4) and a moveable, non-verbal robot (Blossom). However, from our post-interaction surveys with witnesses and users showed no significant differences for EMAR V4 or Blossom. However, on all item responses, participants reported EMAR V4 responses as slightly higher than Blossom. See Figure 5 for more detail.

### 5.4. Categorizing Wizard Responses and Associated Outcomes

In an overall analysis of all teen-robot interactions including V4 and Blossom, there were several distinguishable categories related to how teens operated the robots. From these interactions, we identified specific responses that led to increased engagement and those that led to disengagement.

#### 5.4.1. V4—Verbal Responses

Teen wizards of V4 had the opportunity to type what V4 said in response to the user's stress. Some of the wizard's responses were very successful at creating engagement or a connection with the user and others were not. For a summary of detailed verbal response themes, see Table 4. In almost every example, offering advice to the user led to disengagement, whereas offering an empathic response led to an emotional connection between the teen and the robot. Teens also seemed to greatly appreciate humorous responses and these often led the user to connect to both the robot and the wizard.

**Table 4:** V4 Operator verbal responses and associated outcomes.

**Theme**	**School**	**Operator utterance**	**Outcome**	**User response**
1. Advice	4	“That's too bad, you should try to study a bit more next time.”	Disengagement	“Thanks robot, I didn't really need advice.”
2. Suggestion	1	“Go listen to some music.”	Connection	“Okay. Thanks robot.”
3. Empathy: Active	4	“People do care, I care.”	Emotional Connection	Touches heart and says, “Thank you. Thanks for hearing me out.”
4. Empathy: Passive	4	“That sucks.”	Emotional Connection	“Yeah, it does.”
5. Humor	1	“I have to deal with miserable kids like you day after day. They should really give me AI so I can help you.”	Engagement	Lots of laughter, connection to robot.
6. Inquiry	3	“Why do you think that was so hard for you?”	Engagement	Further discussion / articulation.
7. Reassurance	2	“I am sure everything is going to be okay.”	Emotional Connection	Sigh, “Thank you.”

#### 5.4.2. Blossom—Movement Responses

**Figure 6:**
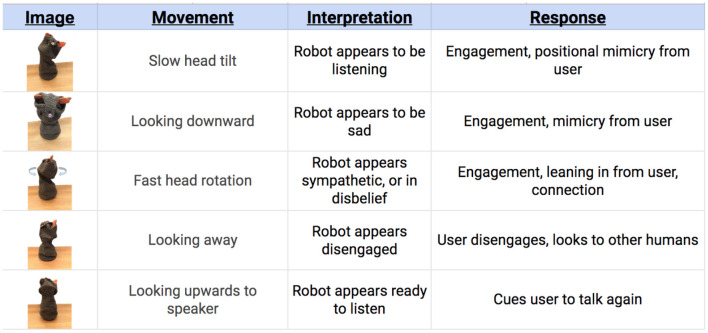
Blossom operator behaviors and associated responses.

Teen wizards of Blossom had the opportunity to use movement as a form of responsiveness to the user's story of stress. Figure 6 illustrates the six main categories of wizard responses and their associated outcomes. Most teens intuitively used a head tilt, or gentle head turning to convey listening or empathy. Rapid head movement with a downward nod, similar to head shaking, was often used to convey disbelief or understanding in relation to a user's stress. Occasionally, teens turned Blossom's head away from the user (sometimes unintentionally) which often signaled looking at witnesses for confirmation. Surprisingly, even when Blossom's movements were not in line with a typical human response, rarely was there any disengagement with the user or the witnesses.

### 5.5. Teen-Robot Interactions

Through the data sources: teen stress stories, open-ended wizard surveys, and interaction video data, we observed teens as operators and the effect their operation had on the users' interaction with the robots. As operators, teens attempted to conduct the robot in an appropriate manner and one that would resonate with their peers. In our analysis of the interactions that occurred as a result of teens operating the two different robots, four key themes emerged: (1) Authenticity, (2) Empathy, (3) Emotional Engagement and (4) Imperfection Creates Connection. These themes were supported by multiple pieces of data captured in our interaction study.

#### 5.5.1. Authentic Operators

Authenticity appeared numerous times in reviewing the open-ended survey and interview data from teens who had operated EMAR V4 and Blossom robots. We saw several examples of teens articulating their attempts to be “real” or “authentic” in their operation of the robot and even in their responses to the robots. One operator of EMAR V4 said, “I tried to say things that I would say to my friend and say things that seemed genuine” (Group interview, School 3). Authenticity also appeared before we had conducted any robot interactions. At our first study site (School 1), we had crafted scripts for the users to read to read the robot in the event they did not want to share their own stress story. Although based in teen data, these scripts were written by our research team. Two teens immediately commented in the margins on the script about our manufactured scripts. One noted, “This is now just making fun of stress rather than dealing with it.” Therefore, we iterated on the method and immediately asked teens to write the actual scripts, verbatim, to use for future interactions. Apparently these teen-written scripts seemed authentic as we never again received negative feedback.

#### 5.5.2. Operating for Empathy

Teen operators were asked to try to make the user feel heard during the interaction. Many teens described attempting to operate the robot in an “empathetic” or “sympathetic” manner. One teen reported, “I was trying to be empathetic, I wish there was more preloaded conversations” [P151, Operator survey, School 4]. As users, teens also expressed feeling empathy from the robot, “The robot didn't say anything, but the movements showed it cared” [P74, Operator survey, School 2]. Overall teens seemed genuinely interested in showing empathy through the robot which often led to an emotional response from the users and witnesses.

#### 5.5.3. Emotional Engagement

The manner in which the teens operated the robots often led to an emotional response from the teen user and sometimes the witnesses.

**Figure 7:**
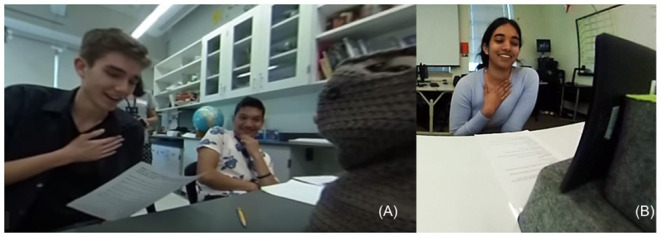
Examples of the *Heartfelt* theme expressed by users during an emotional interaction with the robot. **(A)** Male participant interacting with Blossom. **(B)** Female participant interacting with EMAR.

As users, many of the teens felt the robots cared about them. Throughout participant interactions with the V4 and Blossom robots, our research team observed participants exhibiting engaged facial expressions, body behavior, and verbal responses. We considered the highest level of engagement to be the combination of all three response types. We witnessed on multiple occasions the “heartfelt” gesture which combined a smile (facial expression), hand across the chest (body behavior), and “Aww…” verbal response (Figure 7).

**Figure 8:**
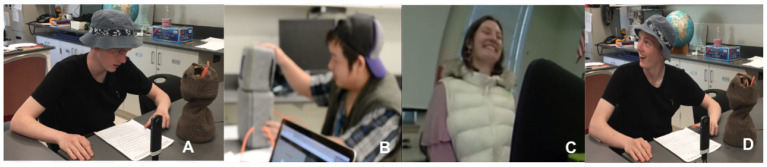
Examples of the emotional responses teens had during robot interaction. Eye contact **(A)**, physical contact **(B)**, laughing **(C)**, social referenceing **(D)**.

Teens often made strong eye contact (Figure 8A) during conversation with the robots. At times, they looked for cues of responsiveness from others (especially in relation to Blossom) before continuing their story. Teens also from time to time touched the robot, not typically during interaction, but in-between interactions or when an interaction was completed (Figure 8B). Many times a users' response to the robot was laughter and engagement (Figure 8C).

Teens also used social referencing (Figure 8D) in response to a particularly salient moment in the robot interaction. During social referencing a teen looks for another teen to acknowledge their experience in that moment. Teens often did this when the robot said something funny, surprising, or truly empathetic. Witnesses were important participants in the interaction. During the study, witnesses were often part of the collective response to the interactions between the wizards and the users interacting with the robot. Witnesses often had similar reactions to the person interacting with the robot. For example, when the wizard had the robot say or do something empathetic, the witnesses would also respond in a similar way to the user interacting the robot. For example, when an operator said “That sucks” in a response to a teens stress story, the witnesses laughed along with the user having the interaction. Further, we observed the witnesses glancing and making eye contact with one another during the robot interactions.

#### 5.5.4. Imperfection Creates Connection

When a novice operator commanded a robot, human error became a part of the human-robot interaction. An EMAR V4 operator would press the return key multiple times and a phrase would be repeated by the robot. A Blossom operator would turn the mobile phone controller too far, causing the robot to spin completely around. These unintended actions, resulting from operator “error” caused unexpected outputs, but interestingly evoked strong engagement (often a response of smiling and laughter) in the user and the witnesses.

In one example, a study participant in the operator role submitted a command to have V4 say, “Wow.” V4 executed the command and spoke out loud, “Wow, wow, wow, wow.” This caused smiling and laughter with all three study participants: the operator, user, and witness. Instead of evoking frustration or irritation from any of the teens involved, they seemed to genuinely enjoy the operator, and thereby robot, imperfections. One might expect that after multiple errors, teens would become frustrated or disengaged, but the opposite seemed to be true in many cases.

Finally, in an exit interview a male participant described Blossom, “I feel like it has a little personality. The way it moves. …even it being a little hard to control makes it seems a little bit real” [Group interview, School 1]. In this example the link between imperfection and realness, suggests that being real or authentic is good and that being fallible is part of that realness.

## 6. Discussion

The breadth of physiological and cognitive responses to robots is challenging to observe without entering more bias into the system. Responses are dynamic, conscious or unconscious, microscopic or macroscopic, and often differ from expressed attitudes. This is why we structured our study to gather attitudinal and behavioral data from multiple viewpoints.

Teens enjoyed participating in this method and found it engaging. And although we found no significant difference between Blossom and V4 measures post-interaction, we did find measurable reduction on the Negative Attitudes Scale, likely resulting from robot interaction.

### 6.1. Authenticity and Imperfection

We repeatedly saw teens attempt to be genuine and authentic operators as well as strong emotional responses from users in both types of interaction. The importance of authenticity for teens is not a new concept (Ullman, 1987; Chessick, 1996). More recently, authenticity has been found an important component in teen health education (Grabowski and Rasmussen, 2014), the success of older teens in college (Lenz et al., 2016), and has been shown to be an integral component of successful mental health counseling for teens (Holliman and Foster, 2016). So it should be no surprise that teens attempted and appreciated authentic behavior authentic behavior, even through the use of a social robot agent.

Robot imperfection has also been studied previously. Mirnig et al. (2017) purposefully programmed faulty behavior into a robot's interaction in order to understand the impact of faults on likability. They found participants preferred the faulty robot interaction significantly more than the flawless interaction. They also showed that shifts in gaze and laughter are typical human reactions to unexpected and imperfect robot behavior. This is very similar to what we saw in our teen-robot interactions.

The teens desire for authenticity, discussed above, might also explain why teen imperfection during robot operation increased engagement and human to human connection during the interactions. Mistakes are human and therefore, reveal the authentic behaviors of humans.

### 6.2. Active Listening

“It could move…it felt more like it was actively listening to you” [P154, School 4].

We identified that successful teen-robot interactions, ones that gained a positive response and strengthened engagement, often included components of active listening. Active listening is an empathetic and therapeutic human to human interaction focused on reflection and empathetic expressions described by Carl Rogers a psychotherapist (Rogers and Farson, 1957). Rogers felt that when people are fully listened to, rather than given advice, or asked to think differently, they can hear themselves more clearly, thus bringing about emotional maturity. The powerful human to human engagement of active listening was the impetus for the first chatbot, Eliza (Rzepka and Araki, 2015) and perhaps a reason that the Eliza program became so engaging for users.

Findings from our study point to the importance of non-verbal and verbal signs of active listening in the human-robot relationship. In the case of non-verbal communication, study participants displayed the strongest emotional connection with the robot when smiling, laughing, and making direct eye contact. These non-verbal communications could be reflected or mirrored by a robot as a sign of attentive listening in future versions of EMAR.

### 6.3. Human to Human Connections

Finally, it is worth noting that in the design and development of our social robot, we have heard concerns from adults (researchers, teachers and colleagues) about the downside of creating a digital agent that teens find truly engaging. The concern raised is often that the robot engagement will mimic that of cell phones and social media which have been suggested as addictive and potentially leading to poor mental health in teens (Twenge, 2019). The main concerns raised by adults about our social robot is that as teens increasingly engage with a digital device, they will further disconnect with the humans in their lives. However, during this interaction study, we see quite the opposite effect. Just as the teen-robot engagement is strong, so are the teen-teen connections during robot interactions. Teens use of social referencing to connect with other teens during interactions and to seek out teen-teen engagement during teen-robot interactions is reassuring that a social robot may be encouraging human-human interaction. This also has been studied in HRI research (Wada et al., 2005; Kim et al., 2013). Finally, teens chose to engage with the robot operator during this study, suggesting that they see through the robot to the operator and can connect with both agents simultaneously. Given that the robot is being designed for a public space and will be interacting with groups of teens, paying attention to the collective responses of the witnesses and the resulting group interactions helps to better understand the communal interaction.

### 6.4. Reflections on the Participatory Wizard of Oz Method

Due to our desire to collect data from the study that was congruent with a human-centered design and participatory approach, we developed a new method of Participatory Wizard of Oz (PWoz). This method has a variety of benefits for collecting data and also some limitations.

In terms of conducting a study, PWoZ offers benefits for participants. First, it provides a level of ecological validity by having the teens themselves develop the scripts and operate the robots for the study. Their actions and choices provide the primary direction and data for the study. Further, making both the constraints and limitations of the robot makes the technology more transparent. The low fidelity nature of the robots are revealed to the participants in the study providing a more realistic impression of the technological capability of the robots, which is quite modest. It both refuses to over promise the ability for social robots to function completely autonomously and also reveals the limitations of the data collected. Second, it provides a more authentic interaction between teens and robots. As evidenced of the teen scripts and the ability to operate the robots, which is more authentic than the adults taking part in the study and making assuming or presupposing what interactions teens might want or find to be authentic. Third, students were highly engaged, and described their participation as fun and enjoyable. The interaction added more humanness into the prospect of designing a robot. Exposing teens to the design of social robots and concepts related to human centered design are promising to engage more young people in STEM related activities and could potential stoke future interest.

In addition to the enjoyable experience of being in the study, in the study, this method provides rich and layered data to inform the design of robots. We used 360° cameras to capture the activity of all the participants in the study: Wizards, users, and Witnesses. This method gives us as much data about the users and witnesses as it does about the Witnesses who are watching the interaction. Further, we can see this data in real time and simultaneously. The layered aspect to this data allows the team to look at the different view points of the interactions. Finally, this approach allows for input from users earlier in the design process, it does not require a fully functional or autonomous robot to get direction from end users about a whole host of considerations for design.

While there were a variety of significant benefits to using this method, there were also limitations to consider and adapt to in the future. There are clear technical limitations given the inability to control the interaction, possibly resulting in a less systematic exploration of possible robot behaviors. Further, this method eliminates the illusion of interacting with an autonomous robot, thus making it difficult to determine how interactions may change once the robot is autonomous.

Implementing this method also proved challenging. First, the tone and directions need to be clearly communicated to participants. We felt fortunate in this study that teens were engaged, cooperative, and interested. This was in part due to the relationship building and partnerships with schools, teachers, and advisors that we had established over time. We could imagine that another group that had not been primed or was less socially connected might pose challenges in having an effective data collection session. Numerous challenges exist when designing and testing social robots in the wild. As discussed by Šabanović et al. (2014) difficulties measuring interactions are compounded by our naturalistic environment, but did offer access to a situated, real-world user experience. Another challenge to doing PWoz in the wild is that each group and each location can provide different challenges. Given that the study was conducted in 4 different locations, sometimes the constraints in each location lead to changes or adjustments in the set up. Finally, while the scripts were written by teens and contained details that were authentic and based on teens experience of their lives, the process of reading them aloud to channel a specific emotion was somewhat artificial. The participants in the study were willing to do this but often did it in a playful way, it was still a simulation. Overall, we found the method to be a promising way to engage teens in the design of a social robot and plan to continue to implement and refine it in future studies.

## 7. Limitations and Next Steps

We chose to conduct all of our studies in the wild (school settings) in order to maintain ecological validity, however, this also meant that many factors were out of our control. For instance, students' operation of or responsiveness to the robot could have been heavily moderated by the room they were in, or the students in their group, as they were self-selected. In addition, our sample was fairly homogeneous given our geographic location and thus, it is important to consider similar studies in other locations, e.g., rural. There may also be cultural aspects (school culture and ethnic cultures) that have influenced our data or how we perceive our data. therefore, continuing to diversify our participant sample and our research team is important. Finally, the teens were greatly limited by the prototype technology and had we presented them with a more robust device, they may have had very different experiences. Teens also did not have much time to become comfortable with the device and the devices were very novel. Both of these factors likely influenced our data and need to be taken into consideration.

## 8. Conclusion

Using social robots to help teens address stress is a promising application. In this study, we privileged the experiences and voices of teens through human-centered design and participatory design to learn more about their needs and preferences for interactions with a social robot prototype. While there were no significant differences between the two social robots that teens interacted with, the rich data collected through the PWoZ method lead to a variety of insights about teens' desires for robots to be authentic, imperfect, and active listeners.

## Data Availability Statement

The datasets for this article are not publicly available due to the identifiable nature of our data and as it incorporates minors. Requests to access the datasets should be directed to the corresponding author.

## Ethics Statement

This study involved human participants and was reviewed and approved by University of Washington Institutional Review Board. Verbal, informed consent to participate in this study as well as the option to consent to the use of a participant's image for research communication purposes was obtained. In addition, written parental/guardian permission for child participation and optional use of child images was obtained in the case of participants who were minors at the time of data collection.

## Author Contributions

EB and MC conceived of the study idea and administered the study with a team of students. MC developed the technology (EMAR V4) specifically for this study. EB created the study design, and analyzed the quantitative data. EB, KT, ER, and MC reviewed all of the data, both quantitative and qualitative, and collaborated to analyze the qualitative data and determine salient findings, and contributed to the writing and revising of the paper.

### Conflict of Interest

The authors declare that the research was conducted in the absence of any commercial or financial relationships that could be construed as a potential conflict of interest.
